# Attitudes toward Nutrition and Dietary Habits and Effectiveness of Nutrition Education in Active Adolescents in a Private School Setting: A Pilot Study

**DOI:** 10.3390/nu10091260

**Published:** 2018-09-07

**Authors:** Samantha Partida, Autumn Marshall, Ruth Henry, Jeremy Townsend, Ann Toy

**Affiliations:** Department of Nutrition and Kinesiology, College of Pharmacy and Health Sciences, Lipscomb University, Nashville, TN 37204, USA; SPartida@mail.lipscomb.edu (S.P.); Ruth.Henry@lipscomb.edu (R.H.); Jeremy.Townsend@lipscomb.edu (J.T.); Ann.Toy@lipscomb.edu (A.T.)

**Keywords:** active adolescents, nutrition knowledge, questionnaire, nutrition intervention

## Abstract

This study was designed to pilot a survey to investigate nutrition knowledge, attitudes, and beliefs toward nutrition, exercise, and dietary habits of active adolescents. Participants included 117 middle school and 40 high school students. General and sport nutrition knowledge, dietary habits, and attitudes toward nutrition education were collected via three electronic surveys. Among middle schoolers, 79.5% of students stated feeling they could benefit from advice about nutrition compared to 92.5% of high school students. The topic scoring the highest in both populations was hydration; the lowest scoring topic category was protein and exercise. Knowledge about healthy eating reportedly comes from parents and coaches most frequently for both high school and middle school students. Less than 40% of students stated their diet meets their nutritional needs. Both middle school and high school students stated a desire to learn more about nutrition, but most nutrition information currently received comes from non-nutrition-related professionals. There is a need for validation of a nutrition knowledge and behavior instrument for United States adolescents, and room for improvement in general and sport nutrition knowledge in active adolescents in all topic areas.

## 1. Introduction

There is a growing interest in sports nutrition in collegiate athletes as a method of improving performance at a high level of competition. However, there are barriers to introducing nutrition education in collegiate athletes who are trying to balance a student schedule with athletic responsibilities. What if nutrition education were introduced at an earlier age and performance enhancing dietary habits began before the athletes started competing at the collegiate level? Until now, multiple studies have acknowledged the lack of nutrition knowledge in adolescents [1,2,3,4,5,6,7,8,9], but few studies have taken the next step in determining how to best educate adolescents on nutrition-related topics.

Many adolescent athletes believe that diet is important for sport performance while also reporting their diet does not currently meet recommended nutritional requirements for sport [5,8]. When incoming collegiate freshman football players were asked about their desire to learn about nutrition related topics, 97% of athletes expressed a desire to learn about nutrition for peak performance, 81% sought to learn about weight gain, and 71% wanted tips on eating out [10]. Even if an athlete believes that nutrition is important for exercise, it might not translate into dietary habits. Specifically, one study found that male high school athletes are more likely to meet nutrition recommendations than female high school athletes despite scoring lower on nutrition knowledge questionnaires indicating that male adolescent dietary practices have less to do with nutrition knowledge and more to do with males eating a higher quantity of food [3]. Further research done on adolescent athletes (aged 15–18 years) has revealed multiple nutrition knowledge deficiencies and indicates that overall dietary habits do not meet nutrition recommendations for sport [11]. Thus, there is a need for nutrition education to extend down to high school and even middle school athletes. Moreover, establishing eating patterns and behaviors in adolescence can result in dietary habits that continue through adulthood [12]; therefore, it is becoming increasingly important to educate adolescents on nutrition and healthy eating patterns, such as the importance of not skipping meals. Previously, analysis of nutrition knowledge has largely been focused on collegiate and elite level athletes. It has been demonstrated that there is a deficiency in general nutrition knowledge among collegiate athletes when translating nutrition knowledge into food choices [13]. In elite athletes, a moderate correlation (*r* = 0.44) was observed in all athletes between dietary habits and nutrition knowledge showing that there is a disconnect between nutrition knowledge and dietary habits and a need for further nutrition education [14]. While it is important to reach these athletes and educate them on the importance of nutrition for their sport and their health, it may be more effective to begin nutrition education in high school and middle school athletes to establish appropriate dietary habits before student athletes reach the collegiate or elite level. Unfortunately, young athletes and their coaches often do not have the nutrition knowledge necessary to impact sport performance or overall health, so there is a need for trained professionals to step in and educate young athletes [15].

Until recently, research on nutrition intervention techniques in adolescents have been sparse despite data indicating there is an interest from adolescent athletes to learn more about nutrition [5,8]. Calella, et al. [16] validated a general and sport nutrition knowledge questionnaire in Italian adolescent athletes in 2017, but there was not a validated assessment tool to measure sport nutrition knowledge in adolescent athletes before then, so data on adolescent nutrition knowledge is very limited [4]. However, now that there are known deficiencies in nutrition knowledge in adolescents, and there is a validated tool to assess knowledge, there is a growing need to determine which education methods are more effective when educating young athletes on general and sport nutrition.

Therefore, the purpose of this study was to analyze the nutrition knowledge of active high school and middle school students in the private school setting, learn more about their attitudes and beliefs toward nutrition, pilot a nutrition knowledge survey, and determine which of three methods of nutrition intervention were most effective in delivering nutrition information to active adolescents.

## 2. Materials and Methods

### 2.1. Participants

In this study, participants were students from a private high school and middle school in central Tennessee. The Institutional Review Board of Lipscomb University approved this study in adolescents with informed consent by the adolescents’ parents. A letter of cooperation was signed by both the principal of the middle school and the principal of the high school allowing researchers into the school for this study. Participants were recruited from mandatory classes in order to get the most accurate representation of the entire student population. At the high school level, students were recruited from four health classes, while middle school students were recruited from six physical education classes. Final participants sampled included 117 middle school students aged 11–15 years, and 40 high school students aged 14–18 years.

### 2.2. Study Protocol

One week before the study started, an explanation of the study was given, and a statement was read informing the students that participation was voluntary. Assent forms were distributed following the recruitment script and all signed assent forms were collected the day they were distributed. Students wishing to opt out of the study were told that they would still have to participate in the study as part of classroom participation, but their data would not be included in the final results. A hard copy of informed consent forms for the parents of students under the age of 18 years were sent home with the students and an electronic copy was sent out by email from the teachers to the parents.

Questionnaires and intervention for this study were broken into sessions in order to accommodate school schedule. On the first day of the study, students were first asked to fill out a demographic questionnaire which included questions about lifestyle-related physical activity and participation in sports. Students were designated as “active” if they self-reported at least 60 min of exercise two or more times a week or participated in school sports two or more times a week. This was chosen as the designation based on the definition of “active” in Calella, Iacullo, and Valerio [16] as those “who practiced sport at least two times in a week.” Non-sport, or lifestyle-related, physical activity was also taken into account loosely based on adolescent (age 6–17 years) physical activity recommendations from the Dietary Guidelines for Americans of at least 60 min a day of moderate to vigorous physical activity [17].

Concurrently, height and weight measurements were taken on the first day of the study. Measurements were taken in a location removed from other students in the classroom. Height was measured with a standard tape measure, with the participant’s back against the wall, standing up straight and without shoes on. Weight was obtained on a scale (Seca^®^813, Seca, Hamburg, Germany) without shoes on, with the participant’s pockets empty and without any heavy jackets since demographic data were collected in the middle of winter.

**Figure 1:**
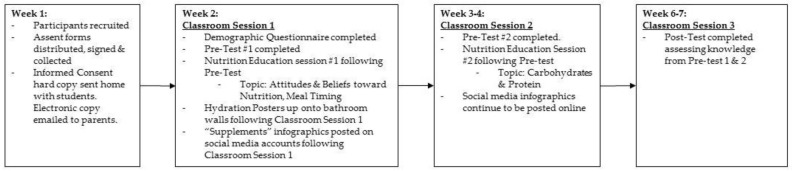
Timeline of events.

Figure 1 shows the timeline of events that occurred throughout the study, including which topics were covered during each nutrition education session.

Following anthropometric data collection, the first day of the study ended with the completion of the first half of the pre-test questionnaire (Pre-Test/Nutrition Education Questions Session #1) which was administered through an electronic survey tool, (Kahoot!© 2018, Oslo, Norway), on student iPads (Apple, Inc., Cupertino, CA, USA) at the middle school and on computers or cell phones at the high school. The survey was projected onto a screen and students answered only as the question was asked to the entire class. This first half of the pre-test included 32 questions over attitudes and beliefs toward nutrition, dietary habits and pre-test nutrition knowledge topics: meal timing, carbohydrates, protein, hydration, and supplements. Questions about attitudes and beliefs toward nutrition were only asked during the first survey and not again during the post-test as researchers were not trying to measure changes in beliefs, but just record attitudes and beliefs toward nutrition at the beginning of the study. Also, questions about dietary habits were asked only during the first survey as dietary habit changes were not being measured in this study. Answers to questions about nutrition knowledge topics were divided between two pre-test questionnaires to be tested again at the end of the study. Classroom nutrition education on meal timing was the only nutrition knowledge topic covered during the first pre-test questionnaire on the first day of the study. Education around exercise was given by a registered dietitian right after questions about meal timing were asked on the first day.

Following the first half of the pre-test questionnaire on the first day of the study in the classroom, poster education on hydration and social media education on supplements was started. Since social media sites Twitter™ (San Francisco, CA, USA) and Instagram™ (Menlo Park, CA, USA) were used to educate about supplements, participants were told to follow the social media handle of the university affiliated with the high school and middle school for nutrition related advice. After all participants had been informed about the account on the first day of the study, social media nutrition infographic posts from the account were posted over the next two weeks. Additionally, following the first day of the study, bathroom posters educating about hydration which included a urine color chart were placed in bathrooms closest to the classrooms and in the locker rooms. Participants in the study were not told that the posters pertained to the study, but posters remained in the bathrooms and locker rooms until the study was complete.

The second day of the pre-test questionnaire was given 1–2 weeks after the first half, depending on classroom schedule and availability. During this session, the second half of the pre-test questionnaire was given. This questionnaire differed from the first in that it covered different topics: carbohydrates and protein. This session was structured so that the researcher surveyed students on 1–3 questions, as pre-test questions, and delivered nutrition education regarding the correct answers to the questions right after all questions were answered. The cycle continued until all questions were answered and explanations were given. This second classroom education session included the second half of the pre-test and the final nutrition intervention in the classroom setting.

The third and final classroom session of the study was a post-test questionnaire on nutrition knowledge about educated nutrition topics: meal timing, carbohydrate, protein, hydration, and supplements. The number of correct answers from the post-test questionnaire were compared to the pre-test answers to determine which nutrition intervention, classroom education, posters in the bathroom or social media education, were most effective.

### 2.3. Questionnaires

The demographic questionnaire used in this study was modified from a questionnaire used in a previous study assessing dietary habits and preferences of 15–18 year-old Irish rugby players [8]. The demographic questionnaire asked questions regarding level of physical activity, participation in sports, level of education of parents, current sources of nutrition education, and most frequent snacks and caffeine sources. Fill-in-the-blank questions were used for level of education of mother/father, participation in sport, position of play, practice schedules and non-sport related physical activity. Multiple answer (i.e., check all that apply) questions were used to ask about current sources of nutrition information, social media accounts used, type of snacks consumed, supplements regularly taken, caffeine usage, and preferences of future nutrition education delivery.

An online survey tool, Kahoot!, was used to record attitudes and beliefs toward nutrition, dietary habits and nutrition knowledge throughout the duration of the study. All online surveys were accessed through school-issued iPads at the middle school, and personal cell phones or computers at the high school level. The first half of the pre-test questionnaire administered on the first day of the study included 32 questions and was modeled after a survey used by Walsh et al. [8] and Calella, Iacullo and Valerio [16]. Questions addressing attitudes and beliefs toward nutrition were asked on a four-point Likert scale and taken from the Walsh questionnaire [8]. Closed, single-answer questions were used to survey students’ current dietary habits and were also taken from the Walsh questionnaire [8]. Finally, true or false questions were used to test knowledge of meal timing before and after exercise, and these questions were modified and taken from the Calella questionnaire [16]. Due to the questionnaire from Calella, Iacullo, and Valerio [16] being so long and asking questions about the same topic in different questions, researchers divided the questionnaire and half of the questions were used for the pre-test and half of the questions into the post-test. The same number of questions on each nutrition topic were included in the pre-test as were included in the post-test. The second electronic questionnaire given on the second day of the study served as the second half of the pre-test and was administered 1–2 weeks following the first pre-test questionnaire. It consisted of 19 closed, single answer questions measuring carbohydrate and protein knowledge prior to nutrition intervention. Questions from this survey were adapted from a previously validated survey of high school students in Italy [16]. The order of the questions was designed so that 1–3 questions were asked and immediately followed by nutrition education about the correct answers to these questions before continuing to the next group of questions. The final 51-question online survey was composed of closed, single-answer questions and served as a post-test over general and sport nutrition topics: meal times, carbohydrates, protein, supplements, and hydration [8,16]. The questions were pre-screened by a high school student and a middle school student for comprehension, but two questions were disregarded in the first class surveyed (10 students) because the answer choices did not match what the question was asking. The answer choices were updated before the survey was used in any other classroom. Researchers used discretion when determining if students were taking the survey seriously. For example, students were eliminated if they were observed randomly choosing answers to questions without reading the question or placing their whole hand on the iPad to randomly choose an answer without looking at the choices.

All questionnaires can be seen in the supplementary material.

### 2.4. Education Interventions

**Figure 2:**
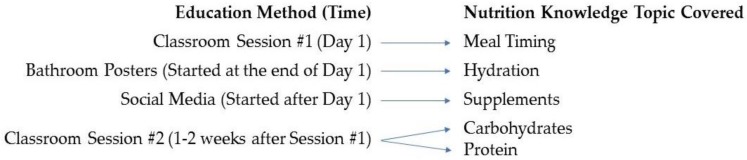
Nutrition education sessions according to the nutrition intervention technique.

Nutrition education interventions were split into three parts throughout the study. Figure 2 shows which nutrition topics were covered during each nutrition intervention. The first nutrition intervention consisted of a Registered Dietitian going into the classrooms of middle school and high school students. This intervention was split into two sessions in order to accommodate school schedule and time.

During the third and final classroom session, no further nutrition education was done, but the post-test on nutrition knowledge was given. The second and third classroom sessions were planned two weeks apart to reduce the possibility that memorization of questions and answers was a factor from the pre-test to the post-test on nutrition knowledge [18]. Following the first classroom education session, social media infographics on supplements (Figures S1–S3) and bathroom posters on hydration (Figure S4) began without a direct explanation given to participants following the first nutrition education session in the classroom. All participants were included in every nutrition intervention, and all participants were given the same pre- and post-test to help researchers determine which nutrition intervention technique was more effective.

Pre-test information was included in baseline data, with or without a complete post-test. The pre-test was given over two different days (classroom session #1 and classroom session #2) in conjunction with nutrition education for the day. On a few occasions, students were present for one day of pre-testing and education but not the other. Post-test data was removed if a student was not educated on the category during the pre-test. Since social media was discussed during the first pre-test session, post-test questions involving supplements from education given on social media were eliminated from post-test data because students were not notified about the social media handle for the information. On one occasion, a student missed both pre-test sessions, so post-test data was not included, but demographic data was included.

### 2.5. Statistical Analysis

Questionnaire scoring was completed by coding correct answers with +1 and using 0 for incorrect, “I don’t know,” or blank answers. Reasons for blank answers include: questions being timed (20+ s) and students’ not responding to questions in time, and potential glitches in the survey software. Dependent *t*-test (matched pairs) was used to test for significant differences between pre- and post-test scores after answers were coded. Statistical significance was determined at an alpha level *p* < 0.05 for two-tailed tests. The software used in this study was JMP Pro 11.0 (SAS Institute Inc., Cary, NC, USA) [19].

## 3. Results

### 3.1. Demographic Data

Students were divided into two groups: active and non-active based on criteria already used by Calella, Iacullo and Valerio [16] and the Dietary Guidelines for Americans recommendation of 60 min a day of physical activity for adolescents age 6–17 years. The general population of high school participants were 59.2% (29) male and 40.8% (20) female. Middle school participants were 49.2% (63) male and 50.8% (65) female. Of those participants, 91.4% of middle school students met the criteria for active, and 83.3% of high school students met the active criteria. Statistical analyses were run on active students specifically. Anthropometric and demographic data on the active students are listed in Table 1. The most common sports reported at the high school were track (8 students), and baseball, basketball and cheer/gymnastics/dance (five students each). At the middle school, basketball (43 students) was the most common sport listed followed by soccer (23 students), volleyball and football (20 students each) and track (18 students). Students were permitted to list more than one sport. The level of education of the parents reported by students indicates that only 3.4% of parents did not have education beyond high school. All parents in this demographic had at least a high school degree, 62.3% had a bachelor’s degree and 34.3% had a higher education degree. Level of education of parents could have an influence on dietary habits and attitude toward nutrition of their children since 93.2% (109 students) of active middle school students and 82.5% (33 students) of active high school students reported getting some of their knowledge about heathy eating from their parents.

### 3.2. Attitude toward Nutrition

In the middle school participants, 79.5% of active students surveyed stated feeling they could benefit from advice about nutrition, while 92.5% of active high school students stated they felt they could benefit. Fourteen of the 40 active, high school participants (35.0%) surveyed feel their current diet meets their nutritional needs while 39.0% of active middle school participants (44 of 117 surveyed) stated their diet meets their nutritional needs. Table 2 indicates an interest among adolescents to learn more about nutrition. When asked on what areas of nutrition the most information is needed, students surveyed reported nutrition and exercise, healthy eating, and suitable snacks as the top three areas of need at both the middle school and the high school. Middle school students reported wanting to receive this nutrition information through coaches most often, while high school students reported wanting nutrition education through information sheets and the internet.

Before nutrition education was initiated, students were surveyed on their beliefs toward nutrition and exercise. Descriptive statistics are displayed in Table 3 showing beliefs in active high school students and active middle school students. Only 22.5% of active high school students reported they think supplements are necessary to support their exercise training program, while 44.3% of active middle school students think supplements are necessary. A greater percentage of active middle school students strongly agreed that increasing muscle mass is essential for sport performance than active high school students (42.5% and 20.0% respectively). The majority of students rated the importance of eating and drinking in exercise performance as important or very important at both the middle school and high school (85.8% and 82.5%, respectively).

### 3.3. Dietary Habits

Dietary habits were surveyed at the beginning of the study during the first pre-test session. The majority of both middle school and high school students ate breakfast every morning (68.8% and 57.5%, respectively), packed a lunch from home (71.7%, 57.5%, respectively), ate a homemade meal for dinner (85.7% and 72.5% respectively) and snacked between meals (87.5% and 97.5%, respectively). Table 4 shows the dietary habits of active middle school and high school students. The most commonly listed snack between meals fell in the chips, popcorn, pretzels, or crackers category. The second most frequently selected snacks were fruits and vegetables.

Dietary habits related to exercise were also surveyed during the first classroom session in conjunction with the first nutrition knowledge pre-test. Water was the main source of hydration in both active middle school and high school students (95.0% and 81.4%, respectively). Table 5 shows the answers to questions regarding fluid intake, meal times before and after exercise, and the food groups of choice before and after exercise.

Finally, supplement and caffeine intake were surveyed to determine what are most commonly consumed by students. Caffeine intake is more prevalent in middle school students than high school. Soda was the most common source of caffeine followed by coffee, and energy drinks and energy shots were the least commonly used sources of caffeine. The most common supplement taken in both groups is a vitamin or mineral supplement. Overall, 57.5% of active high school students and 56.4% of active middle school students reported taking a supplement of some sort (Table 6). For the purposes of this study, supplements were listed as: protein supplements, herbal products, vitamin/minerals, meal replacements, and creatine.

### 3.4. Nutrition Knowledge

Active adolescents’ knowledge about healthy eating reportedly comes from parents and coaches most frequently for both high school (82.5% and 80.0%, respectively) and middle school athletes (93.2% and 73.5%, respectively). When ranking sources of nutrition knowledge, “education program at school” ranked fourth with high school athletes and eighth with active middle school students.

Table 7 shows the remaining pre- and post-test scores on general and sports nutrition knowledge in all categories for active students in both high school and middle school. Post-test scores were not included if education on topics was not received, so there are fewer post-test scores than pre-test scores. The topic on which students scored the highest in both groups on the pre-test was hydration (64.7% and 73.9%, respectively). The lowest scores were on questions over protein and exercise for the middle school and high school students (28.5% and 33.8%, respectively). Both active high school and active middle school students significantly improved on questions about exercise and protein knowledge (*p* = 0.0033 and *p* = 0.0074, respectively). In an earlier survey question, active students were asked in what areas of nutrition the most information was needed, and 56.4% of middle school and 50.0% of high school students stated nutrition and exercise was an area in which they needed more information.

Active high school students significantly improved knowledge in questions on overall general nutrition and on fiber when educated in a classroom lecture setting (*p* = 0.0328 and *p* = 0.0390, respectively). There was no significant difference in scores for middle school students in these categories; however, scores on carbohydrates did decline significantly from the pre- to post-test in active middle school students (*p* ≤ 0.0001) even though carbohydrates were also discussed in classroom lectures.

### 3.5. Education Method Effectiveness

Eighty-four percent of high school students and 77% of middle school students surveyed stated they saw the hydration education posters (Figure S2) in the bathroom and/or locker rooms; however, there was no significant improvement in pre- and post-test hydration knowledge in either group. In fact, hydration scores declined significantly among active high school students (*p* = 0.0026) indicating that the posters were seen but behavior did not change.

Baseline data on social media usage was collected at the beginning of the study, and Instagram was the most commonly used form of social media with 85% of high school students using it and 61.5% of middle schoolers using it. Other forms of social media used include Snapchat™ (Los Angeles, CA, USA) followed by Twitter™ at the high school level, and Pinterest™ (San Francisco, CA, USA) followed by Snapchat™ at the middle school level. At the beginning of the study, only one student surveyed already followed the social media handle used in this study. At the end of the study, only 9.7% of high school students and 10% of middle school students stated they followed the suggested social media handle. The first infographic (Figure 1) posted was titled “Food Before Supplements” and covered sources of nutrition in food form compared to a supplement. No high school students reported seeing this infographic during the study and 14 active middle school students (13.7%) reported seeing it. The second infographic (Figure 2) entitled “Electrolytes” was seen by 4 (12.5%) active high school students and 11 (10.9%) middle school students. The third and final infographic (Figure S1) titled “Vitamins and Minerals” was posted at the exact same time as the second infographic, but only 1 high school student and 21 (21.2%) middle school students reported seeing it. Knowledge on vitamins and minerals in active high school students, which was provided through social media education only, significantly increased in active high school students (*p* = 0.0089). A greater percentage of middle school students than high school students reported seeing the social media graphics, but the mean knowledge scores on dietary supplement usage declined significantly in middle school students (*p* = 0.0010).

## 4. Discussion

In this study, only 35.5% of questions about protein and exercise were answered correctly by active high school students, while 28.5% of questions were answered correctly by active middle school students prior to nutrition intervention. Misconceptions about protein and exercise topics were consistent with findings from similar studies [20]. Rosenbloom, et al. [21] found 47% of male collegiate athletes and 43% of female collegiate athletes believed that protein was the main source of energy for muscle, while 35% of male collegiate athletes and 34% of female collegiate athletes in this study reportedly believed protein supplements were necessary for sport performance. Scores on protein and exercise questions were lowest in both active middle school and high school students; however, significant improvements on the post-test indicates that protein and exercise is a topic about which many active students are interested in learning more. This information also demonstrates that active middle school and high school students acknowledge the connection between nutrition and exercise. For this question and all other nutrition knowledge questions, the difference in the number of students between active students and non-active students was not large enough to compare the two groups, but active students were included in the general population when comparing to the general population. It is possible there were not even enough non-active students in the general population to cause a significant difference. If the general student population is representative of the sample taken in this study, then the general student population is overall active.

In this study, scores on hydration questions were the highest with 77.9% of questions about hydration being answered correctly in high school students and 69.4% of questions being answered correctly in middle school students. In a survey of Egyptian athletes similar in age (13–18 years), 62.6% of those surveyed thought dehydration could affect performance, so there is room for improvement across a widely diverse group of students in regards to nutrition knowledge on hydration [22]. These findings were consistent with hydration questions given to collegiate athletes about exercise and performance, fluid replacement and using thirst as an indicator of hydration status [21].

In collegiate athletes, reported supplement usage over the years has most commonly included creatine, vitamins and minerals, and carbohydrate/electrolyte drinks [20,23]. Creatine use in college athletes is higher than that reported in active high school and middle school students in this study. First of all, level of education about supplements at the high school and middle school athletes is low as seen by a lack of nutrition knowledge in all categories. In the case of the private school used in this study, some of the coaching staff do not agree with the use of creatine as a performance supplement, so creatine use could be lower in this high school population than other school populations. In young Egyptian athletes (age 13–18 years), the most commonly reported supplements used included sports drinks, creatine, vitamins and minerals and amino acids, in that order [22]. As seen in Table 6, results from this study indicate the most commonly used supplements among active middle school and high school students in a private school setting were vitamins and minerals first (46.2% and 45.0%, respectively) and then meal replacements and protein supplements second. The high prevalence of vitamin and mineral usage could be attributed to the socio-economic status of a family that can monetarily afford to put their children through private school. Out of all parents surveyed at both the high school and middle school, only 6 listed a high school diploma as their highest level of education. The other 96.6% of responses regarding parents’ level of education included bachelor’s degree or higher.

Results from this study and other studies regarding the connection between nutrition knowledge and dietary habits indicate that there are many more factors besides nutrition knowledge that impact dietary habits. Students can have all of the nutrition knowledge they need to understand what they need to eat to improve sport performance, but will they do it? Will a student living at home with his/her parents make a change to dietary habits that stray from the norm in the family? Worsley [24] looked into the reason why improving nutrition knowledge does not result in food behavior change and found many different factors contributing to food behavior. Aside from the benefits of eating to improve sport performance, consequences must also be addressed. For example, what will a student have to give up to change his/her dietary habits when eating with friends or family? Will a student have to cook his or her own meals? Do students have the resources and skill to do so, if needed? Adolescents are largely influenced by their environments. Socially, is it “cool” to carry around a water bottle all day long to stay hydrated? Attitudes and beliefs are also factors in this mix of influences on food choices. Does the student believe that the additional effort required to change dietary habits is going to pay off in the end? Finally, after a nutrition education session is over, will the student be self-motivated enough to continue on his or her own? Without immediate gratification, will a student remain motivated to continue? All of these “influences on food behaviors” proposed by Worsley [24] play a role in dietary habits regardless of a student’s level of nutrition knowledge.

Demographic data was collected through a paper survey given before nutrition education began, but all other questioning about attitudes and beliefs toward nutrition, dietary habits and nutrition knowledge were collected through an electronic survey called Kahoot!. Researchers were able to use this electronic tool to record all answers given for each individual participant using their school identification number as a log in. Students were familiar with this tool in “game mode” but that mode was not used in this study. Researchers did have to further modify the original questionnaire in order to fit the character limit required by Kahoot! and students were not allowed to choose multiple answer choices. Where a few questions asked to “check all that apply,” in the original survey, students were asked to choose the single, best option. For example, students were only allowed to select one food group before and after exercise. This could have contributed to some of the differences in food choices at the middle school level. However, the active high school student’s dietary habits stayed consistent from pre-test to post-test. Further research needs to be done on best method of gathering and recording nutrition information data.

High school participants in this study were pulled from health classrooms, so nutrition education was done in the health class while the students were sitting in desks and the survey tool was projected onto a screen. Students used a cell phone or computer to interact with the survey. The learning environment in the middle school setting was different because students were pulled from their physical education classes. Students sat on the floor of the gym with their iPads and the survey tool was projected onto a brick wall. This environment was not conducive to students’ focus on answering the questions. Projecting onto the wall also made some students comment that they had seen the infographic before, but it is possible that the image was not clear and/or bright enough to be well seen, so students mistakenly thought they had seen it before. More students reported seeing a social media infographic than followed the social media account where the infographic was posted. Social media did not turn out to be effective because researchers were unable to get students to follow the University social media account. The low number of students following the social media handle where nutrition education was posted is inconsistent with the significant increase in scores. Further research needs to be done on the use of social media as a method of delivering nutrition education in this age group.

The questionnaire largely used in this study was originally validated on Italian high school students by Calella, Iacullo and Valerio [16] and totaled 62 questions on nutrition knowledge. For the purpose of this study, researchers determined that 62 questions on nutrition knowledge, not including questions regarding attitudes and beliefs or dietary habits, was too many, so modifications to the survey were made to split the survey in half as part pre-test and part post-test. Additionally, the survey was not validated at all in middle school students and, therefore, validation is needed on a survey for that age group. The average age of the active high schooler was 16.18 ± 0.958 years and the active middle school students in this study were 12.37 ± 1.01 years, while the age of the adolescents surveyed to validate the survey in the Calella, Iacullo, and Valerio [16] study were 16.63 ± 1.57 years. The active middle school students in this study were well below the average age of those used to validate the survey, and this may have had an impact on nutrition knowledge scores. The questionnaire was also modified to meet food preferences for American high school and middle school students despite Calella and colleagues stating that the questionnaire could be used in other countries because Italian foods were not specifically used. Changes made include small clarifications such as “brown bread” listed on the original survey being used to explain wheat bread. Modifications were made to both the Walsh, Cartwright, Corish, Sugrue and Wood-Martin [8] and Calella, Iacullo, and Valerio [16] questionnaires to be applicable to all students including active and non-active students since researchers were going into the classrooms and educating all students regardless of activity level. For example, “training day” was changed to “exercise” to include those students not participating on a sports team. Further research is needed on nutrition intervention in students participating in sport.

## 5. Conclusions

Both active middle school and high school students stated a desire to learn more about nutrition, but most of the nutrition information currently received comes from non-nutrition related professionals. Since active students are currently getting their nutrition information from parents and coaches first, further research is needed on the education of students but also the education of parents and coaches to relay reliable nutrition information to students. There is room for improvement in general nutrition knowledge and sport nutrition knowledge in active adolescents in all topic areas, but there is also a need to understand the disconnect between nutrition knowledge and its translation, or lack of translation, into dietary habits.

This study found that educational posters were not read and were not an effective method of delivering nutrition education. Social media was also not effective because students did not choose to follow the required social media handle for this project. Further research needs to be done on the best way of engaging students using social media because the majority of students surveyed revealed that they are using social media and do often get nutrition information from the web. In this study, the most effective method of delivering nutrition education was through classroom lecture, but further research is needed on the best environment of delivering nutrition education, that is, in the weight room versus in the classroom versus in a gym. Finally, students seemed most interested in the relationship between protein and exercise since scores in this category significantly increased in both the active middle school and active high school populations. Overall, further research of nutrition knowledge, attitudes and beliefs toward nutrition, and nutrition intervention in active high school and middle school students needs to be done.

## Figures and Tables

**Table 1:** Demographic data characteristics of active high school and middle school students.

	High School	Middle School	
ANTHROPOMETRICS	(*n* = 40)	(*n* = 117)	
Age (years)	16.2 ± 1.0	12.4 ± 1.0	
Height (cm)	175.6 ± 10.4	159.8 ± 10.6	
Weight (kg)	68.0 ± 14.5	52.9 ± 15.8	
GENDER			
Male, years (% population)	24 (60.0%)	57 (48.7%)	
Female, years (% population)	16 (40.0%)	60 (51.3%)	
GRADE LEVEL			
Freshman	1	6th Grade	45
Sophomore	18	7th Grade	38
Junior	12	8th Grade	34
Senior	9		
LEVEL OF EDUCATION OF PARENTS (question asked for both parents, only answered responses included)			
	(*n* = 48)	(*n* = 127)	
High School	2	4	
Bachelors	23	86	
Masters	14	24	
PhD/MD/JD	9	13	

**Table 2:** Current sources of nutrition information and interest in learning about nutrition.

	High School, *n* (%)	Middle School, *n* (%)
My knowledge about healthy eating comes from: (Check all that apply)		
Education program at school	17 (42.5)	33 (28.2)
Education program at other place	6 (15.0)	30 (25.6)
Teacher	14 (35.0)	35 (29.9)
Parents	33 (82.5)	109 (93.2)
Coach	32 (80.0)	86 (73.5)
TV	15 (37.5)	48 (41.0)
Web	21 (52.5)	45 (38.5)
Friends	13 (32.5)	34 (29.1)
Social Media	13 (32.5)	36 (30.8)
Other	5 (12.5)	10 (8.6)
I have no knowledge	3 (7.5)	6 (5.1)
Do you feel you could benefit from advice about nutrition?		
Yes	37 (92.5)	93 (79.5)
No	1 (2.5)	21 (18.0)
No Answer	2 (5.0)	3 (2.6)
If yes, what areas do you think you need most information on? (Check all that apply)		
Losing Weight	9 (22.5)	36 (30.8)
Nutrition & Exercise	20 (50.0)	66 (56.4)
Gaining Weight	7 (17.5)	18 (15.4)
Healthy Eating	22 (55.0)	60 (51.3)
Suitable Snacks	19 (47.5)	55 (47.0)
Recipes & Cooking	12 (30.0)	34 (29.1)
How would you like this dietary information delivered? (Check all that apply)		
Info Sheets	19 (47.5)	34 (29.1)
Internet, Web Sites	18 (45.0)	38 (32.5)
Info Talks	13 (32.5)	23 (19.7)
School Magazine	0 (0.00)	10 (8.6)
Group Discussion	16 (40.0)	25 (21.4)
Through Coaches	15 (37.5)	54 (46.2)
Other	0 (0.0)	10 (8.6)

**Table 3:** Nutrition and exercise performance beliefs.

	High School, *n* (%)	Middle School, *n* (%)
How do you rate the importance of what you eat in exercise performance?		
Very important	13 (32.5)	58 (51.3)
Important	20 (50.0)	39 (34.5)
Of some importance	5 (12.5)	12 (10.6)
Of no importance	2 (5.0)	4 (3.5)
How do you rate the importance of what you drink in exercise performance?		
Very important	19 (47.5)	47 (41.6)
Important	14 (35.0)	45 (39.8)
Of some importance	6 (15.0)	15 (13.3)
Of no importance	1 (2.5)	6 (5.3)
Increasing muscle mass is essential to sport performance.		
Strongly agree	8 (20.0)	48 (42.5)
Agree	23 (57.5)	53 (46.9)
Disagree	9 (22.5)	9 (8.0)
Strongly disagree	0 (0.0)	0 (0.0)
I think supplements are necessary to support my exercise training program.		
True	9 (22.5)	50 (44.3)
False	17 (42.5)	15 (13.3)
I don’t know	14 (35.0)	48 (42.5)

**Table 4:** Dietary habits of active high school and middle school students.

	High School, *n* (%)	Middle School, *n* (%)
In the past week, how often did you eat breakfast?		
Everyday	23 (57.5)	77 (68.8)
3–5 days per week	8 (20.0)	13 (11.6)
2 days or less per week	5 (12.5)	10 (8.9)
Never	4 (10.0)	12 (10.7)
In the past week, what type of lunch did you eat most days?		
Packed lunch from home	23 (57.5)	81 (71.7)
I bought lunch outside of school	3 (7.5)	4 (3.54)
School lunch	10 (25.0)	27 (23.9)
I didn’t have lunch at all	4 (10.0)	1 (0.9)
In the past week, what type of dinner did you eat most days?		
Homemade meal	29 (72.5)	96 (85.7)
Take-out or fast food	5 (12.5)	6 (5.4)
Meal at a restaurant	5 (12.5)	8 (7.1)
I didn’t have dinner	1 (2.5)	2 (1.8)
Do you snack between meals?		
Yes	39 (97.5)	98 (87.5)
No	1 (2.5)	14 (12.5)
What types of snacks do you eat between meals? (Check all that apply)		
Cakes, sweets, pastries	8 (20.0)	27 (23.1)
Chips, popcorn, pretzels, crackers	31 (77.5)	85 (72.7)
Fruit or veggies	26 (65.0)	77 (65.8)
Protein bars	18 (45.0)	58 (49.6)
Yogurt, cheese, milk	11 (27.5)	63 (53.9)
Cereal bars	8 (20.0)	28 (23.9)
Smaller meals	10 (25.0)	29 (24.8)

**Table 5:** Dietary habits and exercise.

	High School, *n* (%) (*n* = 40)	Middle School, *n* (%) (*n* = 117)
Which of the following fluids do you usually drink during exercise?		
Water	38 (95.0)	92 (81.4)
Soft drinks	0 (0.0)	2 (1.8)
Fruit Juice	0 (0.0)	4 (3.5)
Sports drinks	2 (5.0)	15 (13.3)
How soon before exercise do you eat?		
Within 1 h before	10 (25.0)	52 (46.0)
Between 1–2 h before	17 (42.5)	42 (37.2)
Between 2–3 h before	12 (30.0)	14 (12.4)
More than 3 h before	1 (2.5)	5 (4.4)
How soon after exercise do you first eat?		
Within ½ an hour after	13 (32.5)	43 (38.1)
Between ½–1 h after	16 (40.0)	36 (31.9)
Between 1–2 h after	9 (22.5)	22 (19.5)
More than 2 h after	2 (5.0)	12 (10.6)
Which group of foods do you make sure to eat before exercise?		
Carbohydrates (i.e., bread, pasta, potatoes)	20 (50.0)	11 (10.2)
Protein (i.e., meat, peanut butter, eggs, fish)	15 (37.5)	65 (60.2)
Fruits and vegetables	5 (12.5)	28 (25.9)
Dairy (i.e., yogurt and milk)	0 (0.0)	4 (3.7)
Which group of foods do you make sure to eat after exercise?		
Carbohydrates (i.e., bread, pasta, potatoes)	9 (23.1)	29 (25.9)
Protein (i.e., meat, peanut butter, eggs, fish)	26 (66.7)	49 (43.8)
Fruits and vegetables	3 (7.7)	22 (19.6)
Dairy (i.e., yogurt and milk)	1 (2.6)	12 (10.7)

**Table 6:** Supplement and caffeine usage.

		High School, *n* (%)		Middle School, *n* (%)
Which nutritional supplements are you currently taking? (Check all that apply)				
Protein supplements		4 (10.0)		12 (10.26)
Herbal products		2 (5.0)		3 (2.56)
Vitamins/minerals		18 (45.0)		54 (46.15)
Meal replacements, i.e., bars and shakes		4 (10.0)		13 (11.11)
Creatine		1 (2.5)		0 (0.00)
Total number taking a supplement		23 (57.5)		66 (56.41)
Check how many times per week you drink the following:				
High School				
Days per week	0 days	1–2 days	3–5 days	7 days
Coffee	28 (70.0)	5 (12.5)	3 (7.5)	4 (10.0)
Energy drinks	40 (100.0)	0 (0.0)	0 (0.0)	0 (0.0)
Soda	21 (52.5)	6 (15.0)	11 (27.5)	2 (5.0)
Energy shots	39 (97.5)	1 (2.5)	0 (0.0)	0 (0.0)
Middle School				
Days per week	0 days	1–2 days	3–5 days	7 days
Coffee	82 (70.1)	27 (23.1)	5 (4.3)	3 (2.6)
Energy drinks	111 (94.9)	3 (2.6)	1 (0.9)	2 (1.7)
Soda	46 (39.3)	42 (35.9)	20 (17.1)	9 (7.7)
Energy shots	113 (96.6)	2 (1.7)	2 (1.7)	0 (0.0)

**Table 7:** General and sport nutrition knowledge pre- and post-test scores.

		High School, %			Middle School, %		
	Number of Questions	Pre-Test (40)	Post-Test (33)	*p*-Value	Pre-Test (117)	Post-Test (102)	*p*-Value
General Nutrition Knowledge—Total	16	51.7%	64.0% *	*p* = 0.0328	47.0%	45.7%	*p* = 0.4648
Vitamins & Minerals	6	40.0%	61.1% *	*p* = 0.0089	46.1%	52.0%	*p* = 0.1737
Carbohydrates	4	54.0%	52.4%	*p* = 0.7980	41.2%	27.9% *	*p* < 0.0001
Fiber	3	54.4%	74.2%*	*p* = 0.0390	48.2%	56.5%	*p* = 0.0922
Protein	3	60.5%	76.3%	*p* = 0.0850	54.4%	47.7%	*p* = 0.2348
Sport Nutrition Knowledge—Total	21	58.6%	55.7%	*p* = 0.3132	48.6%	47.4%	*p* = 0.2895
Dietary supplement usage	3	68.1%	54.6%	*p* = 0.0548	50.7%	38.7% *	*p* = 0.0010
Hydration	5	77.9%	64.2% *	*p* = 0.0026	69.4%	67.1%	*p* = 0.1577
Energy & Refueling	7	54.3%	50.8%	*p* = 0.5253	47.0%	45.1%	*p* = 0.3728
Protein & Exercise	6	35.5%	52.7% *	*p* = 0.0033	28.5%	38.4% *	*p* = 0.0074
Total Questionnaire Score	37	56.0%	59.3%	*p* = 0.5872	48.0%	46.7%	*p* = 0.2687

* *p* < 0.05, two-tailed.

## Supplementary Materials

The following are available online at http://www.mdpi.com/2072-6643/10/9/1260/s1, Figure S1: Food over supplements infographic for social media nutrition intervention, Figure S2: Electrolytes infographic for social media nutrition intervention, Figure S3: Vitamins and minerals infographic for social media nutrition intervention, Figure S4: Hydration poster for educational poster nutrition intervention.

## References

[1] Chapman P., Toma R.B., Tuveson R.V., Jacob M. (1997). Nutrition knowledge among adolescent high school female athletes. Adolescence.

[2] Cupisti A., D’Alessandro C., Castrogiovanni S., Barale A., Morelli E. (2002). Nutrition knowledge and dietary composition in Italian adolescent female athletes and non-athletes. Int. J. Sport Nutr. Exerc. Metab..

[3] Douglas P.D., Douglas J.G. (1984). Nutrition knowledge and food practices of high school athletes. J. Am. Diet. Assoc..

[4] Heaney S., O’Connor H., Michael S., Gifford J., Naughton G. (2011). Nutrition Knowledge in Athletes: A systematic review. Int. J. Sport. Nutr. Exerc. Metab..

[5] Manore M.M., Patton-Lopez M.M., Meng Y., Wong S.S. (2017). Sport nutrition knowledge, behaviors and beliefs of high school soccer players. Nutrients.

[6] Nascimento M., Silva D., Ribeiro S., Nunes M., Almeida M., Mendes-Netto R. (2016). Effect of a nutritional intervention in athlete’s body composition, eating behaviour and nutritional knowledge: A comparison between adults and adolescents. Nutrients.

[7] Philippou E., Middleton N., Pistos C., Andreou E., Petrou M. (2017). The impact of nutrition education on nutrition knowledge and adherence to the Mediterranean Diet in adolescent competitive swimmers. J. Sci. Med. Sport.

[8] Walsh M., Cartwright L., Corish C., Sugrue S., Wood-Martin R. (2011). The body composition, nutritional knowledge, attitudes, behaviors, and future education needs of senior schoolboy rugby players in Ireland. Int. J. Sport Nutr. Exerc. Metab..

[9] Wiita B.G., Stombaugh I.A. (1996). Nutrition knowledge, eating practices, and health of adolescent female runners: A 3-year longitudinal study. Int. J. Sport Nutr..

[10] Jonnalagadda S.S., Rosenbloom C.A., Skinner R. (2001). Dietary practices, attitudes, and physiological status of collegiate freshman football players. J. Strength Cond. Res..

[11] Hassapidou M.N., Valasiadou V., Tzioumakis L., Vrantza P. (2002). Nutrient intake and anthropometric characteristics of adolescent Greek swimmers. Nutr. Diet..

[12] Mikkilä V., Räsänen L., Raitakari O.T., Pietinen P., Viikari J. (2005). Consistent dietary patterns identified from childhood to adulthood: The cardiovascular risk in Young Finns Study. Br. J. Nutr..

[13] Dunn D., Turner L.W., Denny G. (2007). Nutrition knowledge and attitudes of college athletes. Sport J..

[14] Harrison J., Hopkins W.G., MacFarlane D.J., Worsley A. (1991). Nutrition knowledge and dietary habits of elite and non-elite athletes. Aust. J. Nutr. Diet..

[15] Cotugna N., Vickery C.E., McBee S. (2005). Sports nutrition for young athletes. J. Sch. Nurs..

[16] Calella P., Iacullo V.M., Valerio G. (2017). Validation of a General and Sport Nutrition Knowledge questionnaire in adolescents and young adults: GeSNK. Nutrients.

[17] U.S. Department of Agriculture (2015). Dietary Guidelines for Americans 2015–2020.

[18] Kline P. (1993). The Handbook of Psychological Testing.

[19] SAS Institute Inc. JMP^®^, 11.0.

[20] Jacobson B.H., Sobonya C., Ransone J. (2001). Nutrition Practices and Knowledge of College Varsity Athletes: A Follow-Up. J. Strength Cond. Res..

[21] Rosenbloom C.A., Jonnalagadda S.S., Skinner R. (2002). Nutrition knowledge of collegiate athletes in a Division I National Collegiate Athletic Association Institution. J. Am. Diet. Assoc..

[22] Tawfik S., El Koofy N., Moawad E.M.I. (2016). Patterns of nutrition and dietary supplements use in young Egyptian athletes: A community-based cross-sectional survey. PLoS ONE.

[23] Wertheimer M. (2013). Eating Behaviors and Supplement Use of College Upperclassmen Athletes versus Lowerclassman Athletes. Master’s Thesis.

[24] Worsley A. (2002). Nutrition knowledge and food consumption: Can nutrition knowledge change food behaviour?. Asia. Pac. J. Clin. Nutr..

