# Supplementary material for: Attitudes toward Nutrition and Dietary Habits and Effectiveness of Nutrition Education in Active Adolescents in a Private School Setting: A Pilot Study
Source: Nutrients. 2018 Sep 7;10(9):1260. doi: 10.3390/nu10091260 (PMC6164939; doi:10.3390/nu10091260)
Supplement: Supplementary file 1 [file nutrients-10-01260-s001.pdf]

Supplementary Materials:

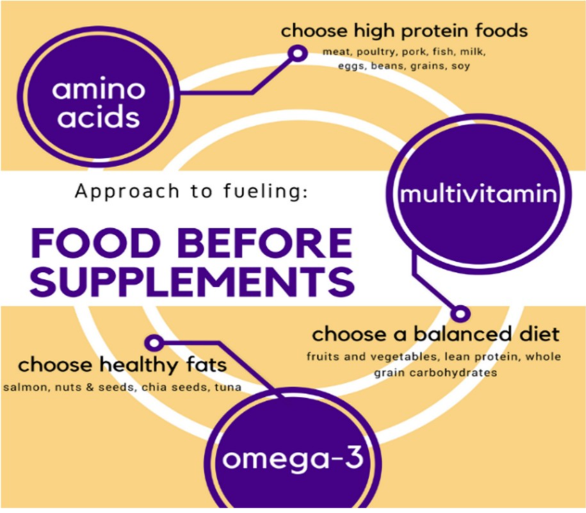

Figure s1. Food over supplements infographic for social media nutrition intervention.

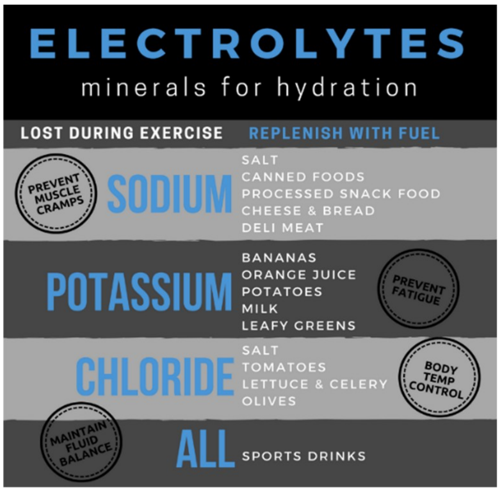

Figure S2. Electrolytes infographic for social media nutrition intervention.

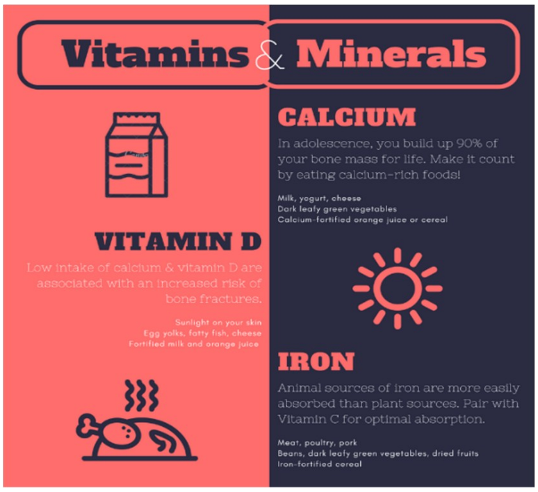

Figure S3. Vitamins and minerals infographic for social media nutrition intervention.

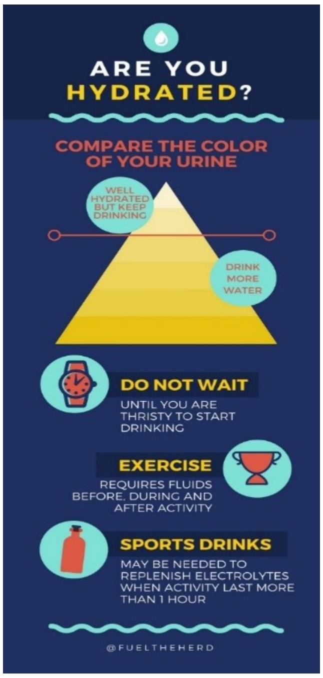

Figure S4. Hydration poster for educational poster nutrition intervention.

A. DEMOGRAPHIC DATA COLLECTION

L-number:
Gender: Male □ Female □
Date of Birth:
Weight: **Height:**
Level of education father/mother:

Do you play sports for your school? Yes □ No □
Sport:
Position:
How many times per week:
Average length (minutes per day):

Non-sport related physical activity? Yes □ No □
How many times per week:
Average length (minutes per day):

My knowledge about healthy eating comes from: (check all that apply)

- □ Nutrition education programs at school
- □ Nutrition education programs in other places
- □ From what my teachers tell me
- □ From what my parents tell me
- □ From what my coaches tell me
- □ Other ______________________________
- □ From what I see on TV
- □ From the web
- □ From my friends
- □ From social media
- □ I have no knowledge about healthy eating

Check the social media accounts that you currently use: (check all that apply)

- □ Facebook
- □ Twitter
- □ Instagram
- □ Snapchat
- □ Pinterest
- □ Other ______________________________

Do you follow Lipscomb University's Sports Nutrition account on Twitter? @FuelTheHerd
Yes □ No □

What type of snack do you eat between meals? (Check all that apply)

□ Cakes, sweets, pastries
□ Chips, popcorn, pretzels, crackers
□ Fruit or vegetables
□ Protein bars
□ Yogurt, cheese, milk
□ Cereal bars
□ Smaller meals
□ Other ____________________

Which nutritional supplements are you currently taking? (Check all that apply)

□ Protein supplements (amino acids)
□ Herbal products (echinacea, ginseng)
□ Vitamins and/or minerals
□ Meal replacements (Slim Fast, protein powders)
□ Creatine
□ Other ____________________

Do you feel you could benefit from advice about nutrition?

Yes □ No □

If yes, what areas do you think you need most information on? (Check all that apply)

□ Advice on losing weight
□ Nutrition and exercise
□ Advice on gaining weight
□ General healthy eating advice
□ Suitable snacks
□ Recipes and cooking skills
□ Other ____________________

How would you like this dietary information to be delivered? (Check all that apply)

□ Information sheets
□ Internet, web sites, links
□ Information talks
□ School magazine
□ Group discussion
□ Through coaches
□ Other ____________________

Check how many times per week you drink the following: (choose one per line)

	0 days	1-2 days	3-5 days	7 days
Coffee or Espresso				
Energy Drinks (i.e. Red Bull, Monster)				
Soda				
Energy Shots (i.e. 5 hour Energy)				

B. PRE-TEST / NUTRITION EDUCATION QUESTIONS SESSION #1

1. Which of the following statements describe you?
   a. I have trouble knowing what I should eat
   b. I feel my diet meets my nutritional needs
   c. I try to follow a diet I believe will improve my performance
   d. I feel pressure from others to follow a particular diet
2. Increasing muscle mass is essential to sport performance.
   a. Strongly agree
   b. Agree

    c. Disagree
    d. Strongly disagree
3. How do you rate the importance of what you eat in exercise performance?
    a. Very important
    b. Important
    c. Of some importance
    d. Of no importance
4. How do you rate the importance of what you drink in exercise performance?
    a. Very important
    b. Important
    c. Of some importance
    d. Of no importance
5. In the past week, how often did you eat breakfast?
    a. Everyday
    b. 3-5 days per week
    c. 2 days or less per week
    d. Never
6. In the past week, what type of lunch did you eat most days?
    a. Packed lunch from home
    b. I brought lunch outside of school
    c. School lunch
    d. I didn't have lunch at all
7. In the past week, what type of dinner did you eat most days?
    a. Homemade meal
    b. Take-out or Fast Food
    c. Meal at a Restaurant
    d. I didn't have dinner
8. Do you snack between meals?
    a. Yes
    b. No
9. Which of the following fluids do you usually drink during exercise?
    a. Water
    b. Soft drinks
    c. Fruit juice
    d. Sports drinks
10. How soon before exercise do you eat?
    a. Within 1 hour before
    b. Between 1-2 hours before
    c. Between 2-3 hours before
    d. More than 3 hours before
11. How soon after exercise do you first eat?
    a. Within ½ an hour after
    b. Between ½-1 hour after
    c. Between 1-2 hours after
    d. More than 2 hours after
12. Which group of foods do you make sure to eat before exercise?
    a. Carbohydrates (i.e. bread, pasta, potatoes)
    b. Protein (i.e. meat peanut butter, eggs, fish)
    c. Fruit and vegetables
    d. Dairy (i.e. yogurt and milk)
13. Which group of foods do you make sure to eat after exercise?
    a. Carbohydrates (i.e. bread, pasta, potatoes)

    b. Protein (i.e. meat peanut butter, eggs, fish)
    c. Fruit and vegetables
    d. Dairy (i.e. yogurt and milk)

14. I think supplements are necessary to support my exercise training program.
    a. True
    b. False
    c. I don’t know
15. Most people can’t get all of the minerals they need from food, so supplements are needed.
    a. True
    b. False
    c. I don’t know
16. You can trust all claims made about supplements, i.e. “This rapidly builds muscle.”
    a. True
    b. False
    c. I don’t know
17. Our bodies create Vitamin D from direct sunlight on our skin when we are outdoors.
    a. True
    b. False
    c. I don’t know
18. The iron in meat is more easily absorbed than iron found in vegetables.
    a. True
    b. False
    c. I don’t know
19. Bread contained a high amount of sodium.
    a. True
    b. False
    c. I don’t know
20. Supplements can be a substitute for an athletes’ meals.
    a. True
    b. False
    c. I don’t know
21. It is better to get vitamins and minerals from supplements than from food.
    a. True
    b. False
    c. I don’t know
22. During exercise longer than 1 hour, sport drinks are better than water (i.e. Gatorade).
    a. True
    b. False
    c. I don’t know
23. You only need to drink when you are thirsty.
    a. True
    b. False
    c. I don’t know
24. Dehydration can reduce exercise performance.
    a. True
    b. False
    c. I don’t know
25. Fluids are needed before, during and after a competition or exercise.
    a. True
    b. False
    c. I don’t know
26. During exercise, you should keep an ice cube in your mouth to drink.

    a. True
    b. False
    c. I don't know

27. Sports drinks and energy drinks are the same thing.
    a. True
    b. False
    c. I don't know
28. Sports drinks contain minerals.
    a. True
    b. False
    c. I don't know
29. After training or exercise,
    a. Wait 2-3 hours before eating to allow time to recover
    b. Eat immediately to start refueling
    c. I don't know
30. You shouldn't eat carbohydrate (i.e. bread, pasta) after 5p or you will gain weight
    a. True
    b. False
    c. I don't know
31. It is necessary to reduce pasta, potato and bread intake during periods of intense exercise
    a. True
    b. False
    c. I don't know
32. It is recommended that an athlete eat a meal rich in carbohydrates 1-2 hours after exercise.
    a. True
    b. False
    c. I don't know

PRE-TEST / NUTRITION EDUCATION SESSION #2

1. The carbohydrate content of ham is:
    a. High
    b. Low or absent
    c. I don't know
2. The carbohydrate content of white bread is:
    a. High
    b. Low or absent
    c. I don't know
3. The carbohydrate content of a tomato is:
    a. High
    b. Low or absent
    c. I don't know
4. The fiber content of honey is:
    a. High
    b. Low or absent
    c. I don't know
5. Wheat bread is richer in fiber than white bread:
    a. True
    b. False
    c. I don't know
6. Bran is the outer part of the grain kernel. This is very high in fiber.
    a. True
    b. False

    c. I don't know
7. The glycemic index of a food:
    a. Indicated the carbohydrate content of a food
    b. Classifies foods based on the blood glucose effect
    c. Indicates blood glucose rise as a result of protein intake
    d. Shows the calorie density of a food
8. Which of the following food choices are good food choices before and after exercise?
    a. Sausage and Chips
    b. Chicken with Pasta and Tomato Sauce
9. You should get most of your energy from carbohydrates when you exercise frequently.
    a. True
    b. False
    c. I don't know
10. Carbohydrate consumption is not good for an athlete
    a. True
    b. False
    c. I don't know
11. The protein content of chicken is
    a. High
    b. Low or absent
    c. I don't know
12. The protein content of a pear is:
    a. High
    b. Low or absent
    c. I don't know
13. The protein content of Parmesan cheese is:
    a. High
    b. Low or absent
    c. I don't know
14. Muscles get most of their energy for exercise from protein
    a. True
    b. False
    c. I don't know
15. If you eat more protein than you need, it is likely to be stored as fat.
    a. True
    b. False
    c. I don't know
16. Meat and eggs contain protein, while other foods do not. These are staple foods for exercise.
    a. True
    b. False
    c. I don't know
17. The more protein you eat, the more muscle you build.
    a. True
    b. False
    c. I don't know
18. Athletes practicing extensive training have double the protein needs of the general population
    a. True
    b. False
    c. I don't know
19. Excessive dietary protein intake can lead to liver and kidney damage.
    a. True

b. False
c. I don’t know

POST-TEST (SESSION #3)

1. In the past week, how often did you eat breakfast?
   a. Everyday
   b. 3-5 days per week
   c. 2 days or less per week
   d. Never
2. In the past week, what type of lunch did you eat most days?
   a. Packed lunch from home
   b. I bought lunch outside of school
   c. School lunch
   d. I didn't have lunch at all
3. In the past week, what type of dinner did you eat most days?
   a. Homemade Meal
   b. Take-out or Fast Food
   c. Meal at a Restaurant
   d. I didn't have Dinner
4. Do you snack between meals?
   a. Yes
   b. No
5. Which of the following fluids do you usually drink during exercise?
   a. Water
   b. Soft Drinks
   c. Fruit Juice
   d. Sports Drinks
6. How soon before exercise do you eat?
   a. Within 1 hour before
   b. Between 1-2 hours before
   c. Between 2-3 hours before
   d. More than 3 hours before
7. How soon after exercise do you first eat?
   a. Within 1/2 an hour after
   b. Between 1/2 - 1 hour after
   c. Between 1 - 2 hours after
   d. More than 2 hours after
8. Which group of foods do you make sure to eat before exercise?
   a. Carbohydrates (i.e. bread, pasta, potatoes)
   b. Protein (i.e. meat, peanut butter, eggs, fish)
   c. Fruit and Vegetables
   d. Dairy (i.e. yogurt and milk)
9. Which group of foods do you make sure to eat after exercise?
   a. Carbohydrates (i.e. bread, pasta, potatoes)
   b. Protein (i.e. meat, peanut butter, eggs, fish)
   c. Fruit and Vegetables
   d. Dairy (i.e. yogurt, milk)
10. If someone fails to meet their protein needs with food, amino acids are necessary.
    a. True
    b. False
    c. I don’t know
11. Dietary supplements are necessary, both in competitive sport and non-competitive.

a. True
b. False
c. I don’t know

12. It is better to get vitamins and minerals from supplements than from food.
    a. True
    b. False
    c. I don’t know
13. Have you ever seen this image? (Food before Supplement)
    a. Yes
    b. No
14. Low calcium and low vitamin D intake are associated with an increased fracture risk.
    a. True
    b. False
    c. I don’t know
15. Our body creates Vitamin D from direct sunlight on our skin when we are outdoors.
    a. True
    b. False
    c. I don’t know
16. Most people will never be able to meet their iron needs with food, so a supplement is required.
    a. True
    b. False
    c. I don’t know
17. Pork and poultry are good sources of iron.
    a. True
    b. False
    c. I don’t know
18. Have you ever seen this image? (Ca, Vit D, Iron)
    a. Yes
    b. No
19. Canned beans are saltier than dried beans.
    a. True
    b. False
    c. I don’t know
20. Sports drinks contain minerals.
    a. True
    b. False
    c. I don’t know
21. Have you ever seen this image? (Electrolytes)
    a. Yes
    b. No
22. Sports drinks are the most appropriate to drink after two hours of exercise.
    a. True
    b. False
    c. I don’t know
23. The best advice for athletes is to drink when they are thirsty.
    a. True
    b. False
    c. I don’t know
24. Important support for the performance of an athlete is water.
    a. True
    b. False

    c. I don’t know

25. Coaches must not allow drinking fluids during an exercise session.
    a. True
    b. False
    c. I don’t know
26. Sports drinks and energy drinks are the same thing.
    a. True
    b. False
    c. I don’t know
27. Have you ever seen this image? (Hydration)
    a. Yes
    b. No
28. At the end of a training session, an athlete must have a meal.
    a. True
    b. False
    c. I don’t know
29. Which of the following food choices is a good choice before and after exercise?
    a. Ham Sandwich with Fruit
    b. Steak and Salad
30. Consuming carbohydrates after 5pm can enhance exercise performance.
    a. True
    b. False
    c. I don’t know
31. It is necessary to increase pasta, potato and bread intake during periods of intense exercise.
    a. True
    b. False
    c. I don’t know
32. It is recommended that an athlete eat a meal rich in carbohydrates 1-2 hours after exercise.
    a. True
    b. False
    c. I don’t know
33. You should get most of your calories from carbohydrates (i.e. bread, pasta, potato).
    a. True
    b. False
    c. I don’t know
34. The carbohydrate content of an apple is:
    a. High
    b. Low or absent
    c. I don’t know
35. The carbohydrate content of cheddar cheese is:
    a. High
    b. Low or absent
    c. I don’t know
36. The carbohydrate content of breakfast cereal is:
    a. High
    b. Low or absent
    c. I don’t know
37. The fiber content of chicken breast is:
    a. Hight
    b. Low or absent
    c. I don’t know
38. Wheat pasta is richer in fiber than white pasta.

a. True
b. False
c. I don't know

39. Bran is the outer part of the grain kernel that is very high in fiber.
    a. True
    b. False
    c. I don't know
40. The glycemic index of food:
    a. Indicated blood glucose rise as a result of protein intake
    b. Indicates the carbohydrate content of a food
    c. Shows the calorie density of a food
    d. Classifies foods based on the blood glucose effect
41. Carbohydrate consumption is not good for an athlete.
    a. True
    b. False
    c. I don't know
42. The protein content of codfish is:
    a. High
    b. Low or absent
    c. I don't know
43. The protein content of chocolate is:
    a. High
    b. Low or absent
    c. I don't know
44. The protein content of rice is:
    a. High
    b. Low or absent
    c. I don't know
45. Muscles get most of their energy for exercise from protein.
    a. True
    b. False
    c. I don't know
46. If you eat more protein than you need, it is likely to be stored as fat.
    a. True
    b. False
    c. I don't know
47. All you have to do to make muscles bigger is eat more protein.
    a. True
    b. False
    c. I don't know
48. Excessive dietary protein intake can lead to liver and kidney damage.
    a. True
    b. False
    c. I don't know
49. Athletes practicing extensive training have double the protein needs of the general population.
    a. True
    b. False
    c. I don't know
50. Chicken contains protein, while other foods do not. This is a staple food for exercise.
    a. True
    b. False

    c. I don’t know

51. Do you follow @FuelTheHerd on Twitter or Instagram?
    a. Yes
    b. No
